# Supplementary material for: G-Protein β-Subunit Gene TaGB1-B Enhances Drought and Salt Resistance in Wheat
Source: Int J Mol Sci. 2023 Apr 15;24(8):7337. doi: 10.3390/ijms24087337 (PMC10138664; doi:10.3390/ijms24087337)
Supplement: Supplementary file 1 [file ijms-24-07337-s001.zip › Figure S1.pdf]

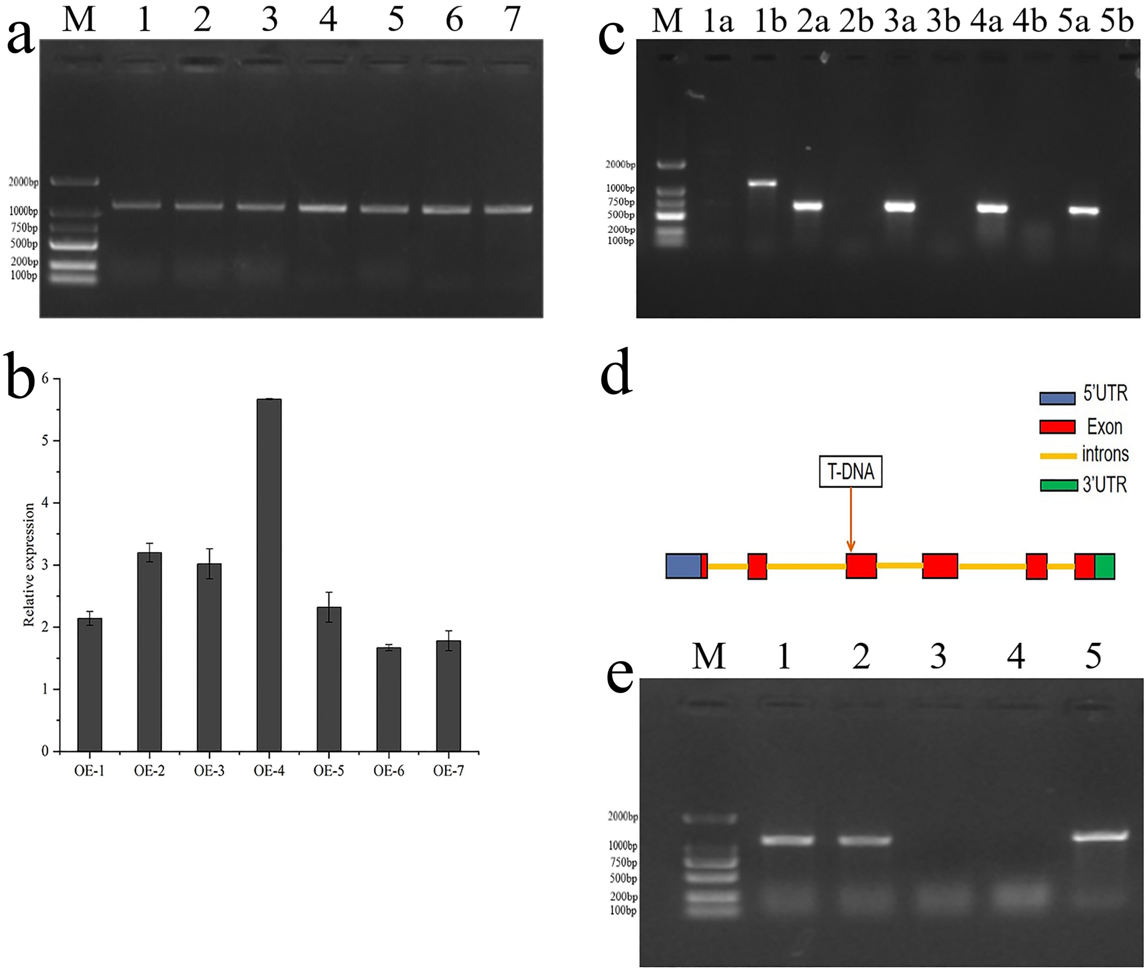

Figure S1. Detection of Arabidopsis lines. (**a**) The positive identification of overexpression Arabidopsis by PCR. M: DL2000; 1-7: different overexpression Arabidopsis lines. (**b**) The positive identification of overexpression Arabidopsis by qRT-PCR. (**c**) Homozygous identification of Arabidopsis mutant *agb1-2*. M: DL2000; 1-5: different Arabidopsis mutant lines. a: BP+RP; b: LP+RP. (**d**) T-DNA insertion diagram. (**e**) Identification of restoring mutant lines. M: DL2000; 1-5: different Arabidopsis restoring mutant lines.
