# Supplementary material for: G-Protein β-Subunit Gene TaGB1-B Enhances Drought and Salt Resistance in Wheat
Source: Int J Mol Sci. 2023 Apr 15;24(8):7337. doi: 10.3390/ijms24087337 (PMC10138664; doi:10.3390/ijms24087337)
Supplement: Supplementary file 1 [file ijms-24-07337-s001.zip › Figure S2.pdf]

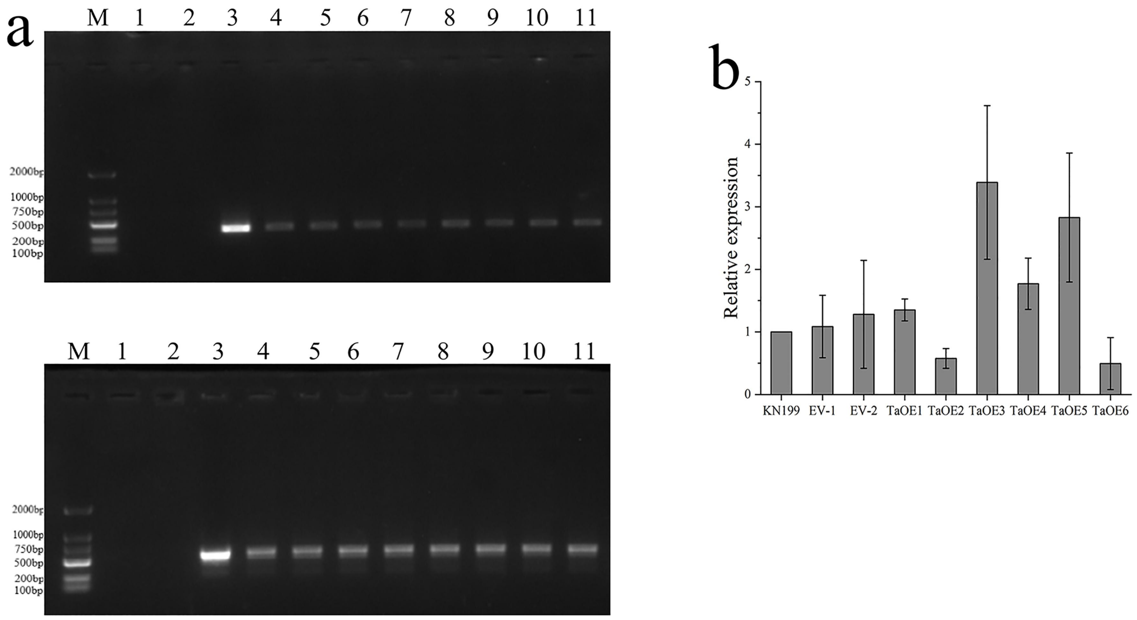

Figure S2. Detection of transgenic wheat lines. (a) The positive identification of transgenic wheat by PCR. M: DL2000; 1: H_2O; 2: KN199; 3: Plasmid; 4-9: Different overexpression wheat lines. 10-11: Transgenic wheat with empty vector gene. (b) The relative expression of transgenic wheat by qRT-PCR.
